# Supplementary material for: Representational structure of perceived food attributes in human occipitotemporal cortex
Source: iScience. 2026 Sep 25;29(10):117660. doi: 10.1016/j.isci.2026.117660 (PMC13635534; doi:10.1016/j.isci.2026.117660)
Supplement: Supplementary file 1 [file mmc1.pdf]

iScience, Volume *29*

Supplemental information

Representational structure of perceived food attributes in human occipitotemporal cortex

Giuseppe Marrazzo, Leonardo Pimpini, Sarah Kochs, Federico De Martino, Giancarlo Valente, and Anne Roefs

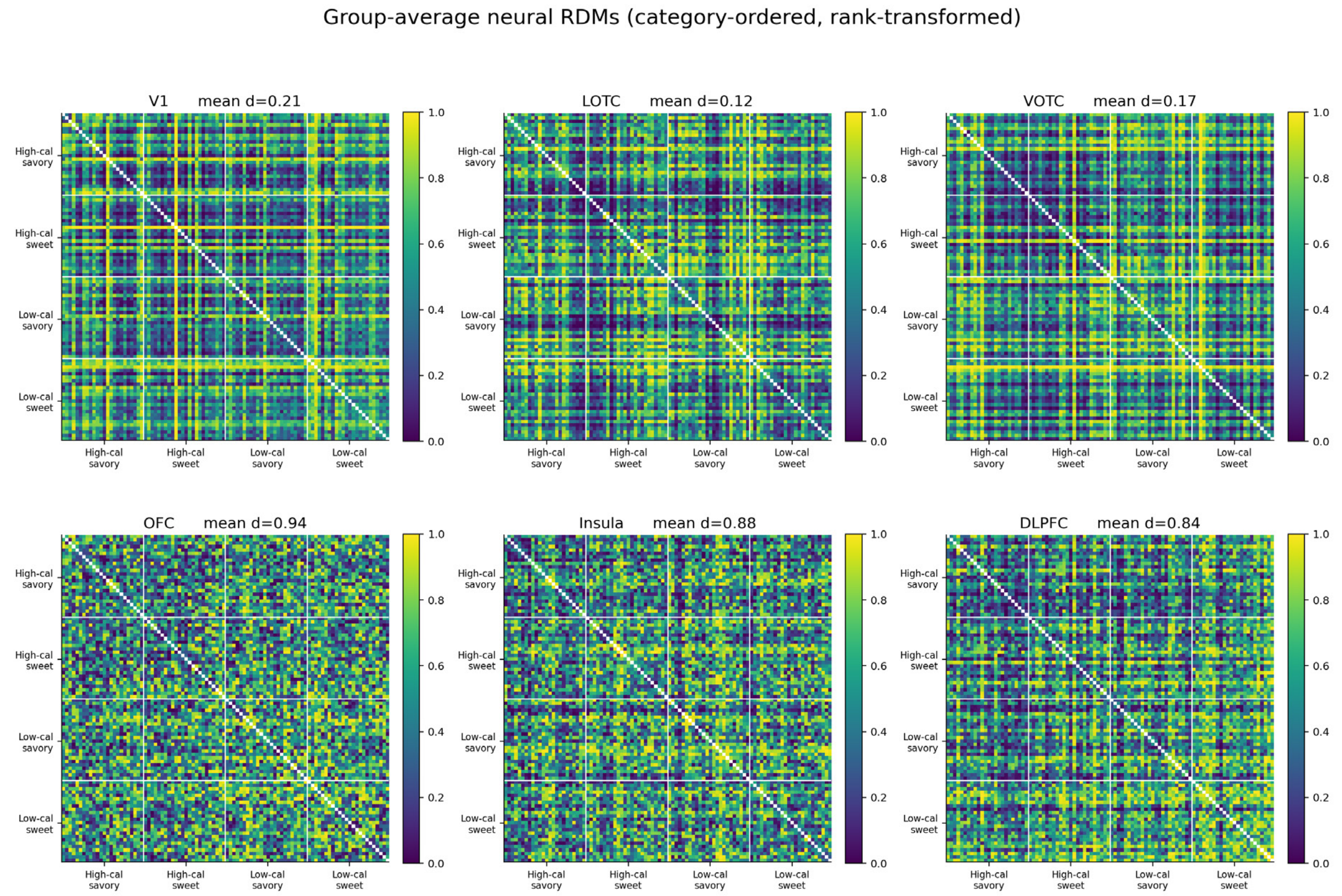

Supplementary Figure S1. Group-average neural RDMs across regions of interest.

Group-average neural representational dissimilarity matrices (RDMs) are shown for V1, lateral occipitotemporal cortex (LOTC), ventral occipitotemporal cortex (VOTC), orbitofrontal cortex (OFC), insula, and dorsolateral prefrontal cortex (DLPFC). For each participant and ROI, neural RDMs were computed from condition-level response patterns across the 96 food stimuli using correlation distance. RDMs were then averaged across participants for visualization and rank-transformed to emphasize relative dissimilarity structure. Stimuli are ordered by the factorial stimulus categories: high-calorie savory, high-calorie sweet, low-calorie savory, and low-calorie sweet. Warmer colors indicate greater neural dissimilarity between stimulus pairs. Mean d indicates the mean rank-transformed dissimilarity value. These group-average RDMs are shown for descriptive visualization only; all statistical inference was performed at the subject level.

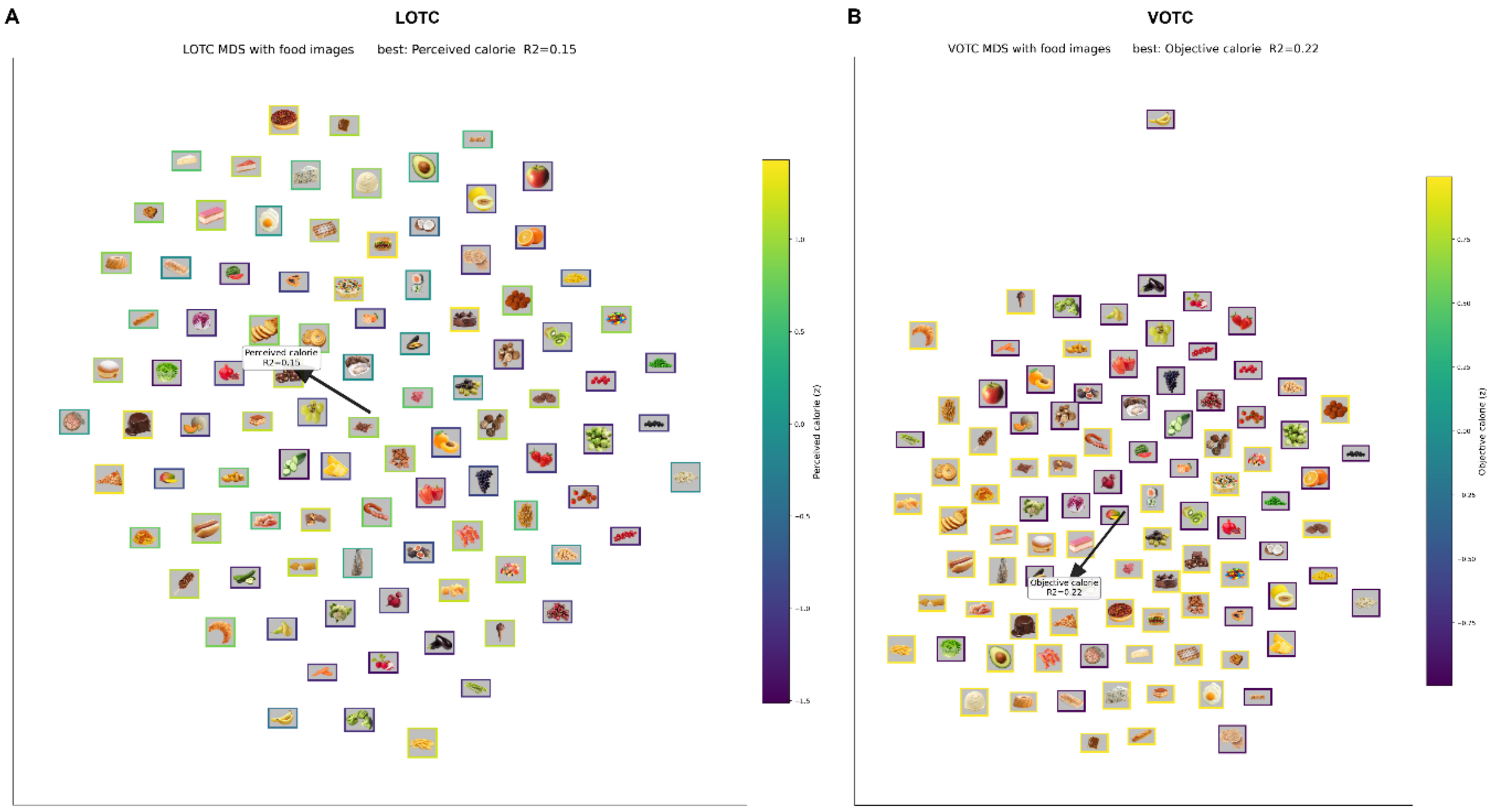

Supplementary Figure S2. Neural MDS visualization of food-stimulus geometry in LOTC and VOTC.

Two-dimensional non-metric multidimensional scaling (MDS) visualizations of the group-average neural representational dissimilarity matrices (RDMs) in lateral occipitotemporal cortex (LOTC; A) and ventral occipitotemporal cortex (VOTC; B). For each participant and ROI, condition-level response patterns were first estimated for each of the 96 food stimuli. Neural RDMs were then computed by calculating the correlation distance between all pairs of stimulus-specific response patterns within the ROI. For visualization, these subject-level neural RDMs were averaged across participants to obtain one group-average neural RDM per ROI. Non-metric MDS was applied to each group-average RDM to obtain a two-dimensional layout in which stimuli with more similar ROI-level response patterns are placed closer together and stimuli with more dissimilar response patterns are placed farther apart. Each thumbnail represents one food stimulus. Thumbnail positions were adjusted slightly after MDS to reduce visual overlap, while preserving the overall geometry of the embedding.

To aid interpretation of the MDS layouts, we fitted simple linear property gradients across the two-dimensional stimulus coordinates. For each candidate stimulus property, including perceived calorie, objective calorie category, palatability, savory/sweet category, and image-color properties, z-scored property values were regressed onto the two MDS coordinates. The property with the highest regression R^2 was selected for visualization in each ROI. Border colors indicate the value of this best-fitting property for each stimulus: perceived calorie in LOTC and objective calorie category in VOTC. Arrows indicate the direction of increasing property values in the MDS space. The reported R^2 values quantify how much variance in the corresponding stimulus property is captured by a linear gradient across the two-dimensional MDS layout. These R^2 values are descriptive visualization metrics and do not represent neural variance explained, RSA model–brain correlation strength, or statistical evidence for a

model effect. All inferential analyses were performed at the subject level using RSA and permutation testing.

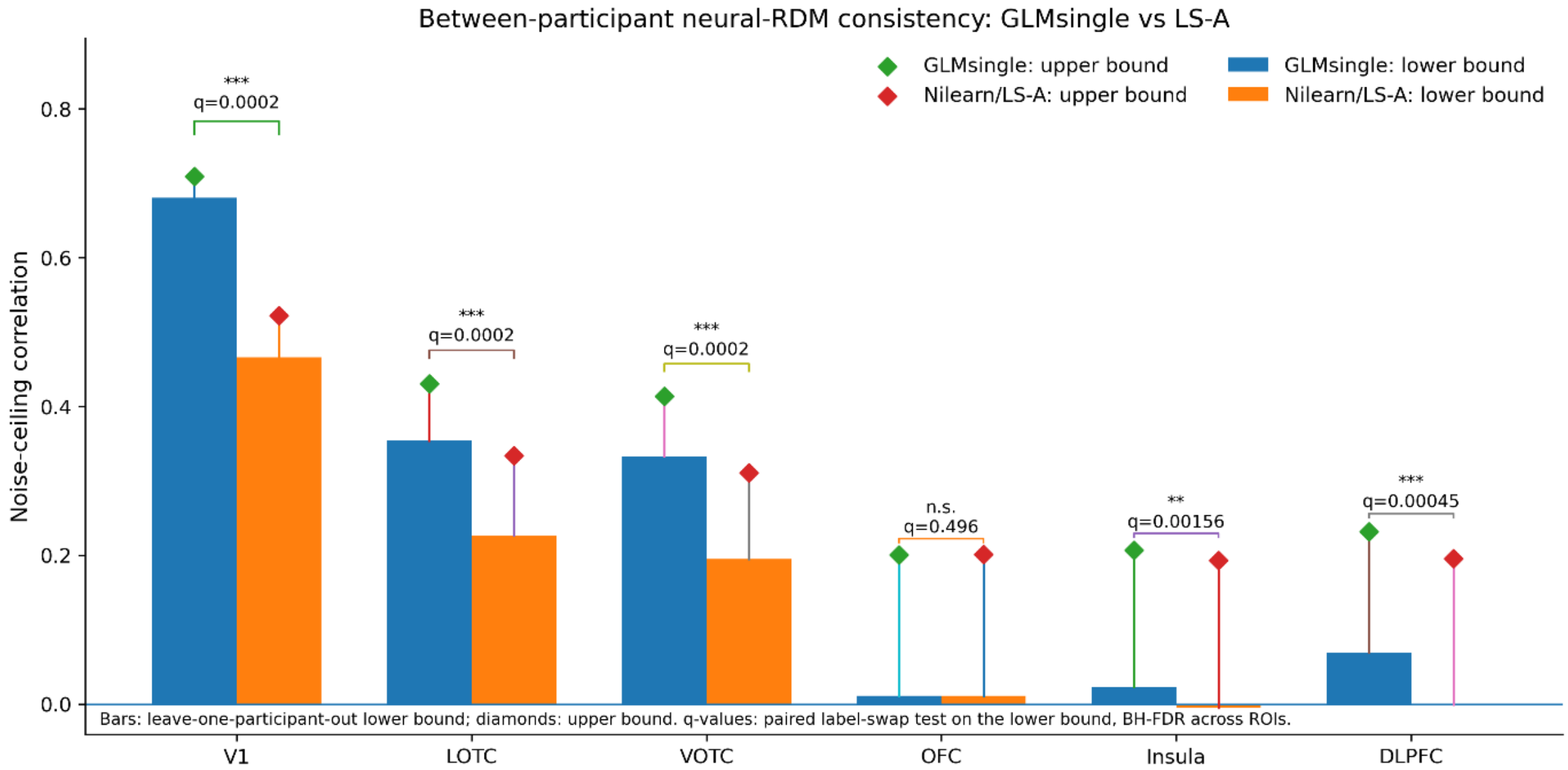

Supplementary Figure S3. Between-participant consistency of neural representational geometry estimated with GLMsingle and LS-A.

Neural representational dissimilarity matrices (RDMs) were recomputed from condition-level response estimates obtained with GLMsingle and with a conventional least-squares-all (LS-A) model implemented in Nilearn [S1]. The two estimation methods were compared within the same participants, stimulus conditions, and ROI definitions. For each participant and ROI, analyses were restricted to the intersection of grayordinates valid under both methods, ensuring that differences in representational consistency could not be attributed to differences in the included feature set. Neural RDMs were constructed from the 96 condition-level response patterns using correlation distance. Noise ceilings were then estimated separately for each method using the between-participant procedure of Nili et al. [S2], with Spearman correlation matched to the main RSA. For each participant, the lower-bound contribution was the correlation between that participant's neural RDM and the group-average RDM computed from all other participants, whereas the upper-bound contribution used the group-average RDM computed from all participants, including the participant being evaluated. The reported lower and upper bounds are the arithmetic means of these participant-level correlations.

Bars show the leave-one-participant-out lower bound, which was treated as the primary measure of between-participant representational consistency because the group reference was estimated independently of the evaluated participant. Diamonds show the corresponding upper bound. Vertical lines connect the lower and upper bounds and do not represent confidence intervals or standard errors.

Differences between GLMsingle and LS-A were assessed using paired within-participant label-swap permutations ($B = 10{,}000$). At each permutation, the complete GLMsingle and LS-A neural RDMs were randomly exchanged within each participant, after which the full lower and upper noise ceilings

were recomputed for the two resulting pseudo-methods. The difference between their lower bounds generated the null distribution expected if the two estimation methods were exchangeable. One-sided permutation p-values tested whether the observed GLMsingle-minus-LS-A lower-bound difference was greater than expected under this null. Benjamini–Hochberg FDR correction was applied across the six ROIs.

GLMsingle yielded higher lower-bound consistency than LS-A in V1 ($q = .0002$), LOTC ($q = .0002$), VOTC ($q = .0002$), insula ($q = .00156$), and DLPFC ($q = .00045$); the difference in OFC was not reliable ($q = .496$). These results indicate greater between-participant reproducibility of stimulus-level neural representational geometry under GLMsingle, rather than establishing estimator accuracy relative to an external ground truth. Asterisks denote FDR-corrected significance: $^{**}q < .01$, $^{***}q < .001$; n.s., not significant.

Supplementary Table S2. Grayordinate feature counts for predefined ROI parcel groups.

The table lists the bilateral Glasser parcels included in the predefined ROI summaries shown in Figure 3A. Left and right HCP-MMP labels were collapsed into bilateral parcel IDs. Feature counts report the number of valid grayordinates retained for RSA after excluding non-finite, all-zero, or zero-variance features. Because the final analysis used all valid grayordinates, the number of valid grayordinates used is the effective feature count for each parcel. Values are shown as mean (minimum-maximum) across participants when they varied across subjects, or as a single value when constant.

ROI group	Parcel	Valid grayordinates used
V1	V1	1618
LOTC	LO1	80
LOTC	LO2	83
LOTC	LO3	125
VOTC	FFC	318
VOTC	VVC	250
OFC	111	323
OFC	131	199
OFC	OFC	680
OFC	pOFC	195.2 (187-196)
Insula	PoI2	333
Insula	MI	224
Insula	AVI	276
Insula	AAIC	139

ROI group	Parcel	Valid grayordinates used
Insula	PoI1	317
Insula	Ig	189
Insula	PI	257
DLPFC	8Av	406
DLPFC	8Ad	481
DLPFC	8BL	341
DLPFC	9p	250
DLPFC	8C	500
DLPFC	p9-46v	309
DLPFC	46	456
DLPFC	a9-46v	221
DLPFC	9-46d	516
DLPFC	9a	282
DLPFC	i6-8	185
DLPFC	s6-8	141

Supplementary Table S3. Summary and rationale for RSA model RDMs and control analyses. The table summarizes the representational dissimilarity matrices (RDMs), derived behavioral variants, and residualized RSA controls used to evaluate visual, subjective, categorical, and controlled accounts of neural representational structure. Continuous behavioral RDMs were constructed from within-participant z-scored ratings. Residualized RSA controls test partial associations in rank-transformed RDM space and are not interpreted as formal variance decompositions.

Model / analysis	Type	What it captures	How it was constructed	Interpretive role
**Gabor**	Low-level visual RDM	Orientation, spatial-frequency, and texture-like image structure.	Pairwise dissimilarities between Gabor feature representations of the 96 food images.	Positive effects indicate neural sensitivity to early image-computable visual structure.

**Model / analysis**	**Type**	**What it captures**	**How it was constructed**	**Interpretive role**
**Color**	Low-level visual RDM	Global color-distribution similarity across images.	Pairwise dissimilarities between Lab color-histogram feature vectors.	Used as a low-level visual control for color-based stimulus similarity.
**CORnet V4**	Intermediate-level visual RDM	Intermediate visual object features approximating mid-level ventral-stream representations.	Pairwise dissimilarities between CORnet V4 feature representations.	Tests whether neural geometry follows mid-level visual structure.
**CORnet IT**	High-level visual RDM	Higher-level object/semantic visual structure approximating later ventral-stream representations.	Pairwise dissimilarities between CORnet IT feature representations.	Tests whether neural geometry follows high-level visual object structure.
**PercPal**	Subjective behavioral RDM	Group-level rating differences for palatability	Within-participant z-scored ratings were averaged across participants to obtain one consensus value per stimulus; pairwise stimulus differences were then computed.	Tests whether neural similarity reflects subjective tastiness/palatability structure.
**PercCal**	Subjective behavioral RDM	Group-level rating differences for perceived calorie content.	Within-participant z-scored ratings were averaged across participants to obtain one consensus value per stimulus; pairwise stimulus differences were then computed.	Primary subjective nutritional model; tests whether neural geometry reflects perceived calorie structure.

Model / analysis	Type	What it captures	How it was constructed	Interpretive role
**PercHealth**	Subjective behavioral RDM	Group-level rating differences for perceived health value.	Within-participant z-scored ratings were averaged across participants to obtain one consensus value per stimulus; pairwise stimulus differences were then computed.	Tests whether neural geometry reflects perceived health structure.
**ObjCal**	Categorical RDM	Predefined high-calorie versus low-calorie category membership.	Binary category-mismatch RDM: pairs from different calorie categories were coded as more dissimilar than pairs from the same category.	Tests whether neural geometry follows objective calorie category rather than graded subjective calorie ratings.
**SavorySweet**	Categorical RDM	Predefined savory versus sweet category membership.	Binary category-mismatch RDM based on savory/sweet stimulus labels.	Tests whether neural geometry reflects the balanced savory/sweet category distinction.
**PercCal/Health/ObjCal | visual controls**	Residualized RSA control	Test the neural association after removing visual-model structure.	Neural and model RDM vectors were rank-transformed and residualized with respect to a visual control RDM such as CORnet IT, CORnet V4, or Color; residual vectors were then correlated.	Positive effects indicate target model neural structure not explained by the specified visual model alone.

Model / analysis	Type	What it captures	How it was constructed	Interpretive role
**PercCal | PercHealth**	Residualized RSA control	Tests the neural association of perceived-calorie structure after removing the component linearly related to perceived-health structure.	Neural and PercCal RDM vectors were rank-transformed and residualized with respect to PercHealth; residual vectors were then correlated.	Positive effects indicate that perceived-calorie structure remained associated with neural representational geometry after removing the component shared with perceived-health structure.

Abbreviations: PercCal = perceived calorie; PercHealth = perceived health; PercPal = perceived palatability; ObjCal = objective calorie category.

Supplementary Table S4. Bayesian evidence for ROI-level RSA effects in higher-order ROIs. Bayes factors were computed on Fisher-z-transformed ROI-level RSA correlations. Mean ρ and SEM are reported on the original correlation scale. *p*-values are uncorrected two-sided one-sample *t*-tests on the Fisher-z-transformed ROI-level effects. BF10 quantifies evidence for a non-zero effect relative to the null; BF01 quantifies evidence for the null relative to a non-zero effect. Evidence labels combine Bayes-factor strength with the sign of the mean Fisher-z effect. Group perceived-calorie, perceived-health, and perceived-palatability models were constructed from group-average z-scored ratings. Subject-own models were constructed separately for each participant from that participant's own ratings.

ROI	Model / contrast	Mean ρ ± SEM	*t*(24)	*p*(two-sided)	BF10	BF01	Evidence
OFC	Group perceived calorie	−0.0001 ± 0.0029	−0.03	.977	0.21	4.74	Moderate null
OFC	Group perceived health	−0.0018 ± 0.0030	−0.60	.555	0.25	4.03	Moderate null
OFC	Group perceived palatability	−0.0053 ± 0.0037	−1.41	.171	0.51	1.97	Inconclusive

ROI	Model / contrast	Mean ρ ± SEM	$t(24)$	p(two-sided)	BF10	BF01	Evidence
OFC	Group perceived calorie | group perceived health	+0.0030 ± 0.0042	+0.70	.492	0.26	3.80	Moderate null
OFC	Own perceived calorie	+0.0023 ± 0.0031	+0.72	.477	0.27	3.74	Moderate null
OFC	Own perceived health	−0.0003 ± 0.0030	−0.09	.932	0.21	4.73	Moderate null
OFC	Own perceived palatability	−0.0027 ± 0.0048	−0.58	.570	0.25	4.08	Moderate null
Insula	Group perceived calorie	+0.0051 ± 0.0037	+1.38	.179	0.49	2.03	Inconclusive
Insula	Group perceived health	+0.0055 ± 0.0038	+1.46	.157	0.54	1.85	Inconclusive
Insula	Group perceived palatability	+0.0105 ± 0.0060	+1.75	.093	0.79	1.26	Inconclusive
Insula	Group perceived calorie | group perceived health	+0.0006 ± 0.0070	+0.09	.933	0.21	4.73	Moderate null
Insula	Own perceived calorie	+0.0019 ± 0.0048	+0.40	.691	0.23	4.41	Moderate null
Insula	Own perceived health	+0.0056 ± 0.0045	+1.24	.228	0.42	2.40	Inconclusive
Insula	Own perceived palatability	+0.0063 ± 0.0057	+1.10	.283	0.36	2.76	Inconclusive
DLPFC	Group perceived calorie	+0.0008 ± 0.0035	+0.22	.828	0.22	4.64	Moderate null
DLPFC	Group perceived health	−0.0089 ± 0.0031	−2.86	.009	5.42	0.18	Moderate negative
DLPFC	Group perceived palatability	−0.0136 ± 0.0070	−1.94	.064	1.05	0.95	Inconclusive
DLPFC	Group perceived calorie | group perceived health	+0.0172 ± 0.0067	+2.57	.017	3.09	0.32	Moderate positive

ROI	Model / contrast	Mean ρ ± SEM	$t(24)$	p(two-sided)	BF10	BF01	Evidence
DLPFC	Own perceived calorie	+0.0044 ± 0.0048	+0.92	.368	0.31	3.25	Moderate null
DLPFC	Own perceived health	−0.0072 ± 0.0034	−2.10	.046	1.37	0.73	Inconclusive
DLPFC	Own perceived palatability	−0.0114 ± 0.0075	−1.52	.142	0.58	1.72	Inconclusive

Note. Positive and negative evidence labels indicate the direction of the observed mean Fisher-z effect. BF10 and BF01 are reciprocal Bayes factors. Conventional descriptive thresholds were used: BF > 3 = moderate evidence; BF > 10 = strong evidence. The contrast "group perceived calorie | group perceived health" tests whether group perceived-calorie structure remains associated with neural representational geometry after removing the component linearly related to group perceived-health structure. Models are shown only for OFC, insula, and DLPFC, the higher-order ROIs examined in this Bayesian sensitivity analysis.

Supplementary Table S5. ROI RSA using participant-specific behavioral RDMs.

For each participant, subject-specific perceived-calorie, perceived-health, and perceived-palatability RDMs were constructed from that participant's own ratings by z-scoring ratings across stimuli and computing pairwise absolute differences. Each subject-specific behavioral RDM was correlated with the same participant's neural RDM within each pooled ROI. Values indicate mean Spearman ρ across participants ± SEM. Directional permutation p-values were computed on Fisher-z-transformed subject-level correlations. $q$within ROI indicates FDR correction across the three subject-specific behavioral models within each ROI. $q$by model indicates FDR correction across ROIs for the same model.

ROI	Subject-specific model	Mean ρ	SEM	$p$perm	$q$within ROI	$q$by model
V1	Own perceived calorie	−0.004	0.004	.841	.999	.841
V1	Own perceived health	−0.015	0.004	.999	.999	.999
V1	Own perceived palatability	−0.021	0.010	.980	.999	.985
LOTC	Own perceived calorie	0.029	0.007	< .001	< .001	< .001
LOTC	Own perceived health	0.032	0.005	< .001	< .001	< .001
LOTC	Own perceived palatability	−0.018	0.009	.966	.966	.985
VOTC	Own perceived calorie	0.030	0.006	< .001	< .001	< .001
VOTC	Own perceived health	0.034	0.006	< .001	< .001	< .001
VOTC	Own perceived palatability	−0.022	0.010	.985	.985	.985

ROI	Subject-specific model	Mean ρ	SEM	*p*perm	*q*within ROI	*q*by model
OFC	Own perceived calorie	0.002	0.003	.241	.720	.362
OFC	Own perceived health	0.000	0.003	.541	.720	.811
OFC	Own perceived palatability	−0.003	0.005	.720	.720	.985
Insula	Own perceived calorie	0.002	0.005	.344	.344	.413
Insula	Own perceived health	0.006	0.005	.114	.219	.227
Insula	Own perceived palatability	0.006	0.006	.146	.219	.875
DLPFC	Own perceived calorie	0.004	0.005	.184	.553	.362
DLPFC	Own perceived health	−0.007	0.003	.978	.978	.999
DLPFC	Own perceived palatability	−0.011	0.007	.927	.978	.985

Supplementary Table S6. Split-half consistency of group-level behavioral rating models.

Split-half consistency was computed across rating participants for perceived calorie, perceived health, and perceived palatability. For each random split, ratings were z-scored within participant and averaged within each half of participants. Consistency was computed both for the stimulus-wise group rating vectors and for the corresponding absolute-difference RDMs used in RSA. Values indicate Fisher-z-averaged Spearman correlations across 10,000 random balanced splits; brackets indicate the 2.5th–97.5th percentile range across splits. Spearman-Brown-corrected values estimate the reliability of the full-sample group model.

Rating dimension	Vector split-half ρ	Vector SB-corrected ρ	RDM split-half ρ	RDM SB-corrected ρ
Perceived calorie	.949 [.933, .962]	.974 [.965, .981]	.938 [.921, .953]	.968 [.959, .976]
Perceived health	.948 [.930, .962]	.974 [.964, .981]	.942 [.924, .955]	.970 [.960, .977]
Perceived palatability	.773 [.675, .840]	.872 [.806, .913]	.532 [.387, .638]	.694 [.558, .779]

Note. SB = Spearman-Brown corrected. Vector consistency reflects the reliability of the stimulus-wise group-average rating axis. RDM consistency reflects the reliability of the behavioral model RDM after converting ratings into pairwise absolute differences and is therefore the more directly relevant reliability estimate for RSA.

References

S1. Abraham, A., Pedregosa, F., Eickenberg, M., Gervais, P., Mueller, A., Kossaifi, J., Gramfort, A., Thirion, B., & Varoquaux, G. (2014). Machine learning for neuroimaging with scikit-learn. *Frontiers in neuroinformatics*, *8*, 14. https://doi.org/10.3389/fninf.2014.00014

S2. Nili, H., Wingfield, C., Walther, A., Su, L., Marslen-Wilson, W., & Kriegeskorte, N. (2014). A toolbox for representational similarity analysis. *PLoS computational biology*, *10*(4), e1003553. https://doi.org/10.1371/journal.pcbi.1003553
